# Management and surgical treatment of parathyroid carcinoma: a 6-year experience of a single centre of endocrine surgery unit

**DOI:** 10.3389/fendo.2023.1278178

**Published:** 2023-11-03

**Authors:** Rita Laforgia, Giovanni Tomasicchio, Federica Cavalera, Maria Sblendorio, Annamaria Spadone, Ferdinando Massimiliano Anelli, Pierluigi Lobascio, Rinaldo Marzaioli, Annunziata Panebianco, Angela Pezzolla

**Affiliations:** Department of Precision and Regenerative Medicine and Jonic Area (DiMePRe-J), Section of Surgery, Laparoscopic and Emergency General Surgery Unit, Hospital University of Bari, Bari, Italy

**Keywords:** parathyroid carcinoma, endocrine surgery, parathyroidectomy, hyperparparathyroidism, PTH - parathyroid hormone

## Abstract

**Background:**

Parathyroid carcinoma (PC) affects 0.1-0.3% of the general population and represents the rarest malignant neoplasms among endocrinological diseases, comprising less than 1%. The best therapeutic treatment and management methods are still debated in the literature. The aim of this study is to evaluate the management and surgical treatment of parathyroid carcinoma after 6 years of enrolment with the Endocrine Surgery Unit of the University Hospital of Bari.

**Materials and methods:**

A retrospective observational study was carried out using a prospectively maintained database of patients affected by primary hyperparathyroidism between January 2017 and September 2022. Consecutive patients over 18 years old with a final histopathological finding of PC were included in the study. Patients with secondary or tertiary hyperparathyroidism, parathyroid hyperplasia, and parathyroid adenoma were excluded. All patients underwent follow-up every 6 months for the first 2 years, and annually thereafter.

**Results:**

In this study, 9 out of 40 patients affected by hyperparathyroidism were included; 6 (66.6%) were female and 3 (33.3%) were male patients, with a median age of 59 years (IQR 46-62). None had a family history of PC. No mortality was recorded while the incidence of recurrence was 22.2%, with a disease-free survival of 8 and 10 months. Parathyroidectomy was performed in five patients, while four patients underwent parathyroidectomy with concurrent thyroidectomy for thyroid goitre. No intraoperative complications were recorded. Open parathyroidectomy was performed with a mini-cervicotomy in seven patients, while two patients underwent robotic surgery. All patients were discharged on the second postoperative day.

**Conclusion:**

PC represents a great challenge in terms of preoperative diagnosis, management and treatment. A surgical approach represents the first best option for PC in referral endocrine surgery units. The early identification of risky patients should be the dominant goal to plan an appropriate therapy and to perform adequate en bloc surgery.

## 1 Introduction

Parathyroid carcinoma (PC) affects 0.1-0.3% of the general population and represents the rarest malignant neoplasms among endocrinological diseases, comprising less than 1% (1). A histological examination is used to detect PC in the majority of cases, because a preoperative diagnosis is difficult due to the absence of specific criteria and fine-needle aspiration is impossible as the violation of the parathyroid capsule could determine subsequent tumour seeding (2–5).

The aetiology of PC is still unknown (6). PC may occur in sporadic cases, especially in those with a single-gland disease, or may be associated with hereditary endocrinopathies such as Multiple Endocrine Neoplasia type 1 (MEN1) or type 2A (MEN 2A), HPT-jaw tumour syndrome (HPT-JT), or familial isolated hyperparathyroidism (FIHP) (7–9).

The increase in parathyroid hormone (PTH) and serum calcium represents clear signs of hyperparathyroidism along with subsequent signs and symptoms. Patients (pts) can be affected by gastrointestinal, neurological, cardiological, bone and kidney diseases, and in some cases, a palpable neck mass. Unfortunately, metastatic disease could affect 10-30% of pts at the time of the diagnosis, where patients present with lung, liver and bone invasion (10).

Differential diagnosis from parathyroid hyperplasia and adenomas could be complex, but the suspicion of cancer increases in cases of certain findings, such as higher levels of serum PTH and calcium, parathyrotoxicosis, and a palpable neck mass. PTH reaches high levels that are 3 to 10 times higher than the normal serum value in cases of PC. In total, 98% of cancers are functioning tumours associated with more frequent bone and kidney disease, while only 2% are characterized by normal serum levels of PTH and calcium along with the evidence of a palpable and invasive mass (11, 12).

Prognosis is poor for PC because of mortality due to uncontrollable levels of hypercalcemia and subsequent diseases. The overall survival rate at 5 years is 78-85%, and at 10 years it is 49-70% (4).

Differential diagnosis from malignant to benign disease is challenging, and the best therapeutic treatment and management methods are still being debated in the literature (13). Several studies have revealed the inefficacy of chemo- and radiotherapy, and further options such as immunotherapy and targeted therapies have been proposed in several studies (11). Concerning surgical treatment, it is mandatory to reach the best oncological result and to also evaluate the gland’s hyperfunction in order to verify whether all pathological tissues were removed or not. In order to predict the eradication of the disease, intraoperative PTH testing is considered as a reliable method, as is the application of two preoperative scores, especially in case of impossibility to verify intraoperative PTH. The CaPTHUS score, described by Kebebew et al. in 2006 (14), and the Wisconsin index (Win score), described by Mazeh et al. (15) in 2013, were applied for predicting single-gland disease with primary hyperparathyroidism by De Pasquale et al. (16).

The aim of this study is to evaluate the management and surgical treatment of parathyroid carcinoma after 6 years of enrolment with the Endocrine Surgery Unit of the University Hospital of Bari. The secondary aim is to compare the results with those from existing studies. PC remains a difficult disease to preoperatively detect and prevent. The early identification of at-risk patients should be the dominant goal to plan an appropriate therapy and to perform adequate en bloc surgery.

## 2 Materials and methods

A retrospective observational study was carried out using a prospectively maintained database of patients affected by primary hyperparathyroidism with suspected PC who, according to the guidelines for the management of primary hyperparathyroidism from the American Association of Endocrine Surgeons (17), underwent surgery in the Endocrine Surgery Unit between January 2017 and September 2022. Consecutive patients over 18 years old with a final histopathological finding of PC were included in the study. Patients with secondary or tertiary hyperparathyroidism, parathyroid hyperplasia, and parathyroid adenoma were excluded.

All patients underwent a physical examination, cervical ultrasound, computer tomography and 99m Tc sestamibi scintigraphy as part of their diagnostic work-up to locate the lesion; F-fluorodeoxyglucose pet/ct was performed only in selected cases.

Gender, age, clinical symptoms at admission and family history, as well as serum PTH, calcium, phosphate, creatinine, and 24-hour urinary calcium and phosphate levels, were recorded preoperatively. Calcium was recorded in mg/dL (normal range 8.4–10.2 mg/dL), PTH in pg/mL (normal range 8.7–79.6 pg/mL), phosphate in mg/dL (range 2.5–4.5 mg/Dl), creatinine in mg/dL (range 0.84–1.21), 24-hour calciuria in mg/kg/24 h (normal value < 4 mg/kg/24 h) and 24-hour phosphaturia in g/L/24h (normal value < 1.35 g/L/24h). The postoperative parameters registered included serum PTH, calcium, and possible surgical complications. All patients underwent follow-up every 6 months for the first 2 years, and annually thereafter. Postoperative hypocalcaemia was considered as a serum calcium value lower than 8.4 mg/dL with a normal PTH value. Postoperative hypoparathyroidism was considered as a serum PTH value lower than 8.7 pg/mL with a calcium value lower than 8.4 mg/dL. Both complications were considered transient if lasting less than six months and definitive if lasting longer. Informed consent was obtained from all patients before enrolment. All investigations complied with the principles of the Declaration of Helsinki.

### 2.1 Statistical analysis

Continuous parameters were reported as medians and IQRs. The categorical variables were recorded as numbers and percentages. Statistical analysis was carried out using RStudio (R version 4.0.3; R Foundation for Statistical Computing, Vienna, Austria).

## 3 Results

In this study, 9 out of 40 patients affected by hyperparathyroidism were included; 6 (66.6%) were female and 3 (33.3%) were male patients, with a median age of 59 years (IQR 46-62). At admission, two (22.2%) patients had nephrolithiasis, four (44.4%) had bone disease, four (44.4%) had chronic kidney failure (where two of them were waiting for kidney transplantation), one had Berger’s disease and three patients (33%) were asymptomatic. None had a family history of PC.

In the preoperative work-up, all patients had a high level of PTH (median 432 pg/mL, IQR 296.5 – 877) and hypercalcemia (median value 11.20 mg/dL, IQR 11-14.30) except for one female patient with a value of 8.8 mg/dL, whose PC was discovered during a total thyroidectomy for a thyroid goitre. Calciuria and phosphaturia were recorded in only two patients. Parathyroidectomy was performed in five patients, while four patients underwent parathyroidectomy with concurrent thyroidectomy for a thyroid goitre. No intraoperative complications were recorded. Open parathyroidectomy was performed with a mini-cervicotomy in seven patients, while two pts underwent robotic surgery. All patients were discharged on the second postoperative day. Postoperative complications included five patients (55.5%) who had transient postoperative hypocalcaemia, which was treated with oral calcium supplementation, and three (33.3%) patients who developed definitive postoperative hypoparathyroidism. Two patients had high postoperative PTH values of 180 and 249 pg/mL, while seven patients were in the normal range. Histopathological examination revealed that all patients had PC with a Ki67 always ≥ 4% (median value 4%, IQR 4-7), while three (33%) patients were chromogranin-positive, and two (22.2%) patients were synaptophysin-positive.

After a median follow-up of 36 months (IQR 12-48), no mortality was recorded while the incidence of recurrence was 22.2% (2 patients) with a disease-free survival of 8 and 10 months. Follow-up included endocrinological, surgical and dental evaluations, a renal US scan, and a biochemical blood test. Two patients had the highest postoperative PTH values of the series, and both underwent parathyroidectomy with uneventful recovery and were discharged on the second postoperative day.

The clinical data of patients with PC are summarized in **Table 1**.

**Table 1:** Clinicopathologic features of parathyroid carcinoma patients.

Gender	Age	Clinical Symptoms	Calcium (mg/dL)	PTH (pg/mL)	Phosphate (mg/dL)	Creatinine (mg/dL)	Phosphaturia (mg/dL)	Calciuria (mg/dL)	Sugery	Calcium Post (mg/dL)	PTH Post (pg/mL)	Definitive hypo- parathyroidism
F	46	neprholithiasis, osteopenia, osteoporosis	11.2	1800	1.9	0.96	38.1	7.3	TT + PrT	10.1	28	Yes
M	65	neprholithiasis, osteopenia, osteoporosis	9.7	180	45	1.65	0	0	Inferior right PrT	10.2	180	No
F	43	CKF	8.8	/	/	14.3	0	0	TT+ PrT	6.3	<6	Yes
F	59	None	14.3	/	/	1.06	0	0	TT+ PrT	6.5	<6	Yes
F	61	osteopenia, osteoporosis, fatigue, dyspesia	11.1	238	3	0.63	546	323	inferior right PrT	7.4	37	No
M	37	osteopenia, osteoporosis CKF, hyperuricemia	17.6	355	/	1.51	0	0	inferior left PrT	8.5	249	No
F	62	CKF, Berger’s disease	14.3	1156	7	12.3	0	0	superior, inferior right and inferior left PrT + TT	5.5	8	Yes
F	57	None	14.1	598	2.1	0.9	0	0	inferior right PrT	9.3	13	Yes
M	63	CKF	11	432	7.5	9.46	0	0	inferior left PrT	6.7	78	No

TT, total thyroidectomy; PrT, parathyroidectomy; PTH, parathyroid hormone; CKF, chronic kidney failure. /, not available.

## 4 Discussion

The best therapeutic option to cure pts affected by PC is a pre- or intraoperative diagnosis and a complete resection of the tumour during the first initial surgical approach.

In this study, nine cases of PC were detected in 6 years, according to the evident percentage in the literature1. The prevalence of female patients is relevant (66,6%), and this result can be important in cases where there is suspicion of PC, considering that there is the same prevalence in primary hyperparathyroidism (18). The median age of patients in this study was 59 years (IQR 46-62), while Machado et al. reported a tendency for a younger age of about 50 years at the time of the diagnosis (11, 18). A certain diagnosis of PC was evident after the histological examination in all cases. All patients presented high serum levels of PTH and calcium.

**Figure 1:**
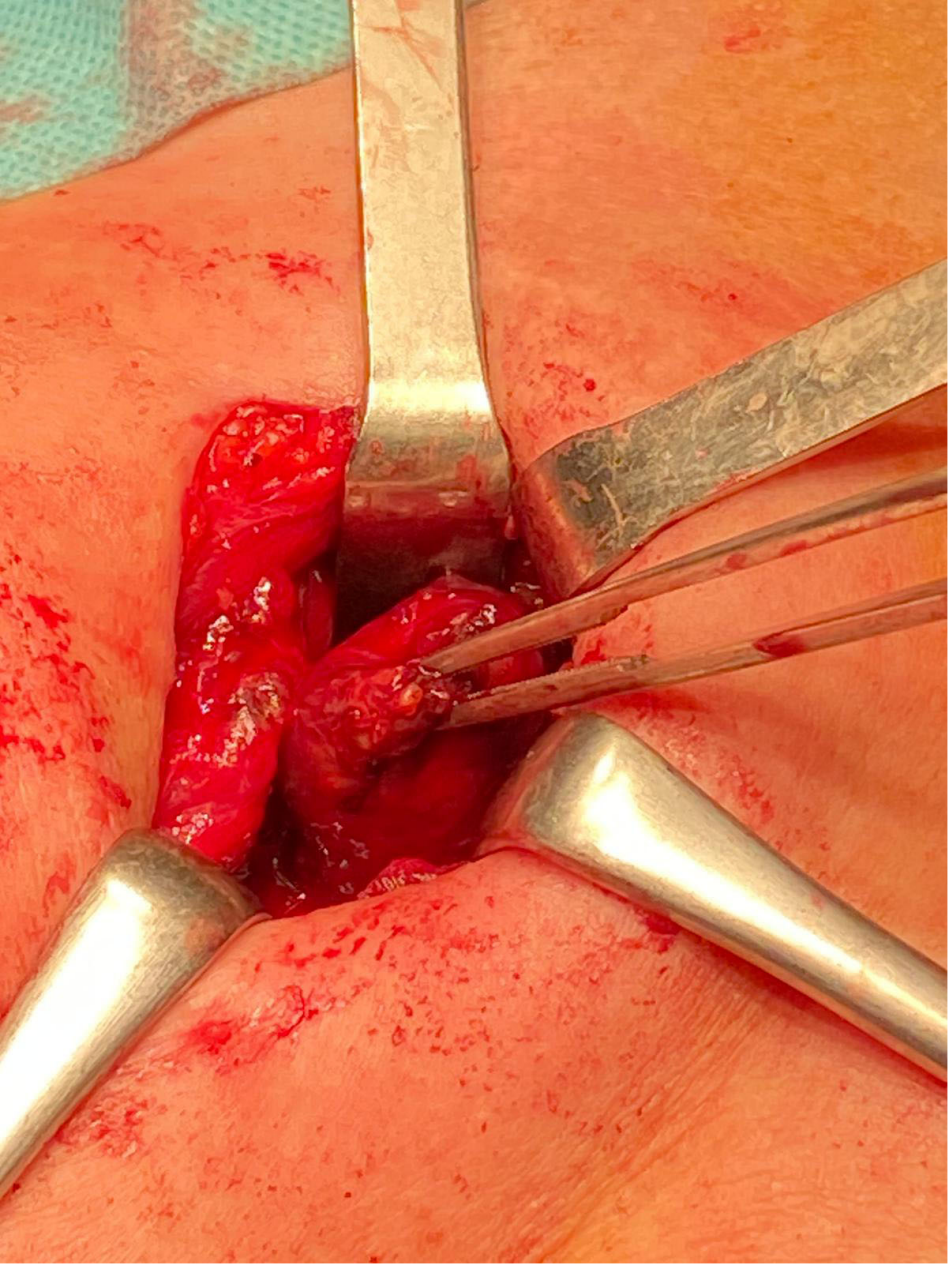
Intraoperative visualization of parathyroid gland.

**Figure 2:**
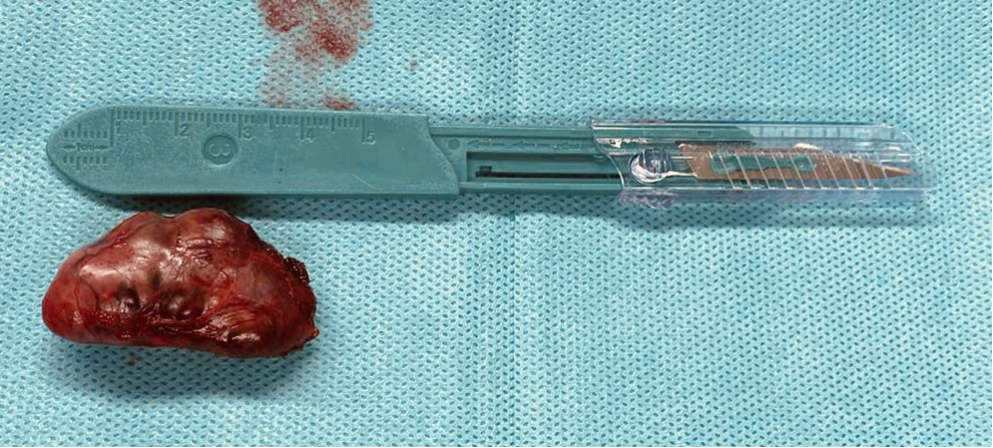
Parathyroidectomy.

The first surgical approach was parathyroidectomy in five patients, with a subsequent secondary surgical step according to a multidisciplinary evaluation; in four cases, thyroidectomy was performed because of a compressive goitre. The same experienced endocrine surgeon performed all procedures, respecting all principles of en bloc surgery: complete exploration, a bloodless field, minimal manipulation of the tumour, evaluation of nodal involvement and exploration of the ipsilateral laryngeal nerve. In all cases, no nodal or nerve involvement was evident. The suspicion of PC could be also intraoperative because the tumour presents with a hard consistency, a fibrous capsule, and a white or grey hue, and it is adherent to nearby structures (**Figures 1**, **2**). Adenomas can appear softer and have a reddish colour with well-defined limits (11, 19).

Two cases of recurrence (22,2%) are still not treated because of a clinical condition not suitable for surgery. According to Cetani et al., 3 years represents the mean time to diagnose recurrence, although some studies reported recurrences after 20 years (2, 6). In cases of recurrences, surgery still represents the best therapeutic option with the main aim of relieving symptoms and eradicating the residual disease.

Several studies have contributed to understanding the natural history, diagnosis, genetics and treatment of PC. Unfortunately, the rarity of this disease, the existing results coming from the few cases that have been reported and a lack of complete clinical data represent the main reason for the absence of adequate guidelines. Moreover, follow-up information is not usually available.

The prevention of PC is difficult and the best expectation is to develop a specific genetic analysis and preoperative diagnosis method. In 2017, Silva-Figueroa et al. (20) proposed a prognostic scoring system to detect PC. The score was based on three risk groups (low, moderate and high) according to three main variables and the recurrence-free survival rate. Although this score is still not validated, it could be associated with clinical, genetic and histological markers in order to realise a preoperative PC diagnosis. In 2021, Shulte et al. (21) distinguished the main histological and clinical features between parathyromatosis, atypical parathyroid adenomas and PC. This study included a strict number of cases and revealed a higher level of cellular necrosis in PC and in adenomas without a clear distinction.

According to the existing results in the literature, the suspicion of PC should be based on severe PTH high levels, hypercalcemia, bone and kidney diseases, and an evident neck mass. The best therapeutic option is still surgery due to the en bloc surgery concepts carried out in referral endocrine surgery units (19, 22–24). Gurrado et al. (25) also suggested that the suspicion of PC should be considered in cases of high values of PTH, hypercalcemia and a mass with an ultrasound diameter that is more than 3 cm.

The role of immunotherapy could be useful to decrease tumour size in isolated cases, as demonstrated by Betea et al. (26). According to Wei and Harari, two cases of inoperable metastatic PC underwent experimental immunotherapy with satisfying results (3).

Genetic and epigenetic investigations are in progress, aiming to identify specific factors and characterize biological molecules and the genetic network of PC cells in order to subsequently develop targeted therapies (12).

## 5 Conclusion

PC represents a great challenge in terms of preoperative diagnosis, management and treatment. Its rarity is not helpful in acquiring clear genetic, clinical and histological features. A surgical approach represents the first best option for PC in referral endocrine surgery units. In this context, in order to offer the best treatment that can optimize clinical management and surveillance, a multidisciplinary team should play a key role. Further multicentric trials are necessary to improve preoperative diagnoses and outcomes, to prevent recurrences, and to recognize at-risk patients.

## Data availability statement

The raw data supporting the conclusions of this article will be made available by the authors, without undue reservation.

## Ethics statement

The studies involving humans were approved by Azienda Ospedaliero Consorziale Policlinico di Bari. The studies were conducted in accordance with the local legislation and institutional requirements. The participants provided their written informed consent to participate in this study. Written informed consent was obtained from the individual(s) for the publication of any potentially identifiable images or data included in this article.

## Author contributions

RL: Conceptualization, Writing – original draft. GT: Writing – review & editing. FC: Data curation, Writing – original draft. MS: Software, Writing – original draft. AS: Data curation, Writing – original draft. FA: Validation, Writing – original draft. PL: Validation, Writing – review & editing. RM: Supervision, Validation, Writing – review & editing. APa: Validation, Writing – original draft. APe: Conceptualization, Writing – review & editing.
